# Safety of oral propranolol for neonates with problematic infantile hemangioma: a retrospective study in an Asian population

**DOI:** 10.1038/s41598-023-33105-2

**Published:** 2023-04-12

**Authors:** Ronghua Fu, Yun Zou, Zhiping Wu, Pingliang Jin, Jun Cheng, Hanxiang Bai, Mengyu Huang, Xiangquan Huan, Hua Yuan

**Affiliations:** Department of Plastic Surgery, Jiangxi Provincial Children’s Hospital, Nanchang, China

**Keywords:** Skin diseases, Paediatric research

## Abstract

Although the efficacy of propranolol in the treatment of infantile hemangioma (IH) has been well established, clinical data on the safety and tolerability of propranolol in neonates are still lacking. In this work, clinical data of 112 neonates with IH were analyzed retrospectively. All of the patients were evaluated in the hospital at the beginning of the treatment and later in outpatient settings during the treatment. Each time, the following monitoring methods were applied: physical examination, ultrasound echocardiography (UCG), electrocardiography (ECG), blood pressure (BP), heart rate (HR), and basic laboratory tests including blood glucose (BG), liver function, blood potassium, thyroid function. There was a significant reduction in BP and HR at the initiation of treatment. The incidences of bradycardia and hypoglycemia were observed to be increased with the prolong duration of treatment, but not prolonged PR interval. During the course of the therapy, the risk of hyperkalemia and hypothyroidism was reached maximum at the 2 months and 3 months, respectively. Physical growth index including average height, weight and head circumference was not influenced by the treatment. The observed adverse effects were majority mild and only 3 patients needed to rest for 7 days due to severe diarrhea before restarting treatment. This study demonstrated that propranolol is safe and well-tolerated by properly selected young infants with IH. No serious adverse events were observed.

## Introduction

With an incidence of 4–10%, infantile hemangioma (IH) caused by the proliferation of vascular endothelial cells is the most common benign vascular tumor during childhood^1^. If not treated in time, these tumors have a characteristic evolution that includes a period of rapid proliferation, regression, and completion of regression. IHs can occur on any body part but primarily develop in the head-face-neck region. Although most of the IHs could regress spontaneously, approximately 70% of children suffer from sequelae, such as telangiectasia, excessive fibrofatty tissue, and skin laxity^2^. Furthermore, about 10% of patients could develop complications, including obstruction, functional impairment, ulceration, and disfigurement^3^. Therefore, such cases are problematic and need prompt consultation with a specialist and early intervention to withdraw the progression of IH.

Historically, the various management options for problematic IHs included intralesional and systemic corticosteroids, chemotherapeutic agents, laser therapy and surgical intervention^4^. Unfortunately, these treatment options have potentially considerable side effects. In 2008, Leaute-Labreze et al. found that propranolol could promote the regression of infantile hemangioma when they applied propranolol in the treatment of a patient with tachycardia complicated by IH^5^. Since then, propranolol has become the main drug for the treatment of IH worldwide. Meanwhile, a growing body of research has further proved that propranolol can prevent growth and induce IH regression, which is more effective and safer than other treatment methods, including corticosteroids^6–10^. Currently, propranolol administration has been considered to be the first-line therapy for problematic IH.

The specific growth characteristics of IHs is that the rapidest and most significant growth occurs at the age of 1–3 months, and most of the growth is completed at the age of 5 months^11^. Further, Tollefson and colleagues reported that the most rapid IHs growth occurs between 5.5 and 7.5 weeks of age, much earlier than previously appreciated^12^. The authors of this study proposed that there is a period of opportunity, prior to the rapid growth, rather than after growth has been accomplished, to treat high-risk IHs and optimize outcomes. Although it is efficacious that propranolol treatment starts at beyond the period of rapid proliferation, some irreversible consequences may have already resulted, such as permanent skin changes. Moreover, current research have shown that early intervention, especially starting at the proliferative stage, is associated with an increased likelihood of excellent results and avoiding functional impairment^13,14^. Thus, for IHs requiring clinical intervention, the optimal timing of starting treatment is either before or as soon as appearing irreversible consequences, including permanent disfigurement or dysfunction.

Nevertheless, the drug propranolol applied to children may cause risks and side effects such as atrioventricular block, bradycardia, hypotension, and hypoglycemia^15^. Pediatricians who are not positive about treating IHs with the drug propranolol, especially for young infants, often emphasize the systemic effects of these drugs, particularly on the cardiovascular system, and argue that the US Food and Drug Administration (FDA) has not approved propranolol for “correcting premature infants < 5 weeks of age”.

Although, premature or young infants with IHs have been reported to successfully treated with propranolol, no comprehensive data regarding the basic laboratory tests, cardiovascular data, physical development and side events was offered^16,17^. Inspired by this, a study to assess the safety of propranolol in young infants was conceived, hoping to provide evidence-based data for future treatment recommendations. The overriding goal of this study was to analyze the safety of initiating and maintaining propranolol for problematic IH in young infants in term of basic laboratory tests, cardiovascular data and physical development.

## Materials and methods

Clinical data of 112 infants with IH admitted from January 2016 to December 2020, Department of Plastic Surgery, Jiangxi Provincial Children’s Hospital, were analyzed retrospectively. The propranolol administration contraindications were excluded by basic laboratory and cardiac examinations. Basic laboratory tests included blood glucose, liver function, thyroid function, renal function, and so on. Cardiac examinations included ECG, UCG. Inclusion criteria comprised: infants younger than 1 month (corrected actual age) at the time of propranolol initiation for treatment of IH. Exclusion criteria included: infants were treated for a diagnosis other than IH or 1 month older at the time of propranolol initiation.

This research was granted by the Institutional Review Committee of ethics Committee of Jiangxi Provincial Children’s Hospital, and all guardians signed informed consents. This study was conducted according to the ethical guidelines of the Declaration of Helsinki.

### Methods

All infants were scheduled to be hospitalized for 24 h monitoring at the first 3 days during the initial propranolol treatment. The initial dose of propranolol administration was twice daily at daily total dose of 0.5 mg/kg for day 1, and then the oral dose was increased to daily total dose of 0.75 mg/kg and 1 mg/kg daily for day 2 and 3, respectively. Patients took the first dose of propranolol at 8:00 a.m. In order to avoid the risk of hypoglycemia, we asked patients to take propranolol within 30 min after feeding. During the outpatient follow-up, oral propranolol was adjusted to daily total dose of 2 mg/kg when patient was older than 1 month.

Hypotension was defined as BP: 0-1 month < 50/30 mmHg, 1–2 months < 60/35 mmHg, 2–3 months < 65/40 mmHg, 3–6 months < 70/45 mmHg, 6–12 months < 75/50 mmHg, 12–24 months < 75/50 mmHg^18,19^. Bradycardia was defined as HR: 0–1 < 110 bpm, 1–2 months < 105 bpm, 2–3 months < 100 bpm, 3–6 months < 90 bpm, 6–12 months < 80 bpm, 12–24 months < 70 bpm^18,19^. Hypoglycemia was defined as BG level < 40 mg/d (2.2 mmol/L) before 1 month of age and < 3.89 mmol/L after 1 month of age^20^.

#### During hospitalization

We arranged for the same nurse to measure BG, HR and BP. Noninvasive multi-parameter monitor was used for continuous bedside monitoring of all infants. HR and BP were measured at 8:00, 9:00, 10:00, 21:00 and 20:00 each day. Fingerstick blood glucose monitoring was used to measure the BG level before the first dose of propranolol (baseline) and 2 h after administration. The oral dose of propranolol was not added on the next day, when infants occurred hypotension or bradycardia.

#### During follow-up

At 1, 2, 3, 5, 7, 10 and 13 months after the beginning of therapy, the parents and children returned to the clinic together. During each follow-up period, if children was still taking oral propranolol, ECG, UCG and basic laboratory tests was performed; otherwise, it was not required.

The height, weight and head circumference of children was measured at the age of 6, 12, 18, 24 and 30 months.

Propranolol was considered to be discontinued gradually in 3 weeks, if IH fully involuted (no lesion was found by ultrasound) or severe side evens occurred. And all of side reactions (such as diarrhea, sleep disturbances, vomiting, agitation) told by parents was documented.

### Statistical analysis

Quantitative data were displayed by means and standard deviation or median and interquartile range. And categorical data were described by number and percentage (N, %). Continuous data were analyzed by independent t-test and categorical data were compared using Pearson’s chi-square test. SPSS v.22.0 statistical software (IBM Corporation, Armonk, NY, USA) was used for statistical analysis.

### Ethical approval

All procedures performed were in accordance with the ethical standards of the institutional research committee (Radboudumc Committee on Research Involving Human Subjects, reference number 2017-3850) and with the 1964 Helsinki declaration and its later amendments or comparable ethical standards. This research was granted by the Institutional Review Committee of ethics Committee of Jiangxi Provincial Children’s Hospital, and all guardians signed informed consents.

### Informed consent

This research was granted by the Institutional Review Committee of ethics Committee of Jiangxi Provincial Children's Hospital, and all guardians signed informed consents.

## Results

### Patient demographics and hemangioma characteristics

112 patients were included in the study and Table 1 shows the characteristics of patients. A total of 80 females and 32 males were included, and the ratio of female-to-male was 2.5:1. The median age and weight at the initiating treatment was, in general, 17 days of life (interquartile range, 13–22 days) and 4.2 kg (interquartile range 3.6– 5.0 kg), respectively. The patients’ median age at the beginning of propranolol treatment was 17 days (interquartile range, 13–22 days). There were fortyone (36.6%) premature infants among this study. The median weight at the start of propranolol administration was 4.2 kg (interquartile range 3.6–5.0 kg). The most frequently location of IH was the head, face and neck area. 36 (32.1%) patients were diagnosed as segmental morphologic subtype in this study.

**Table 1:** Baseline characteristics of patients and IH. ECG, electrocardiogram; IH, infantile hemangioma.

Characteristics	Value (%)
Patients	112
Gender*	
Male	32 (28.6)
Female	80 (71.4)
Gestational age*	
Term born (≥ 37 weeks)	71 (63.4)
Born prematurely (< 37 weeks)	41 (36.6)
Age at treatment (corrected actual age, day)**	17 (13–22)
Weight (kg)**	4.2 (3.6–5.0)
ECG findings*	
Normal	105 (93.8)
Abnormal	7 (6.2)
Congenital heart defects*	
Yes	11 (9.8)
No	101 (90.2)
IHs	
Location*	
Head, face and neck	71 (63.4)
Extremity	23 (20.5)
Trunk	18 (16.1)
Morphologic subtype*	
Localized	53 (47.3)
Segmental	36 (32.1)
Indeterminate	21 (18.8)
Multifocal	2 (1.8)

*Values are presented as a number (percentage).

**Values are presented as a median (interquartile range).

Seven patients among this cohort had abnormal ECG manifestations, 4 of them had nonspecific intraventricular conduction delay, and 3 had right bundle branch block. Eleven patients were confirmed to have congenital heart diseases via UCG, including atrial septal defect (5 patients), ventricular septal defect (3 patients), patent ductus arteriosus (1 patient), mild coarctation of the aortic (1 patient), mild pulmonary valve stenosis (1 patient). The defects found in UCG scans were considered not absolute contraindications for treatment with propranolol, after a consultation with a cardiologist.

### Monitoring of patients during the hospitalization

#### BP and HR

**Figure 1:**
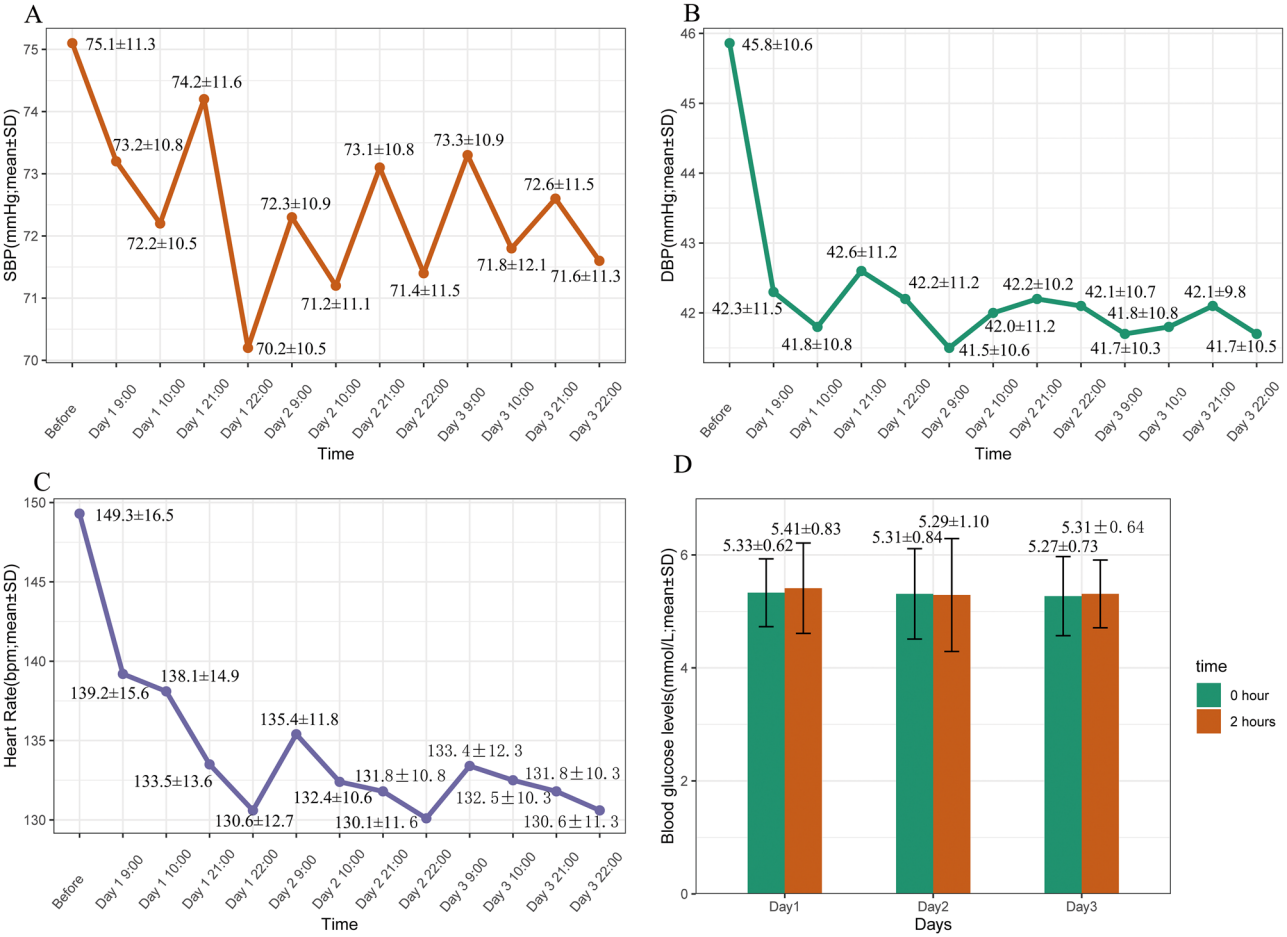
Changes of hemodynamics and blood glucose after propranolol during the hospital. Mean systolic blood pressure (SBP), mean diastolic blood pressure (DBP) and mean heart rate (HR) changes after administration (**A–C**). Mean blood glucose levels before and after 2 h of propranolol at day 1, 2 and 3 (**D**).

Two patients did not add the oral dose in the first month, due to the drop in BP. Compared with 1st hour after propranolol initiation, the systolic blood pressure (SBP) of infants was lower in second hour after propranolol. Infants’ diastolic blood pressure (DBP) was significantly lower than prior to propranolol initiation. The change trends of SBP and DBP over time are shown in Fig. 1, which were statistically significant (P = 0.015, P < 0.001, respectively).

Four patients did not increase the oral dose on the second day because of bradycardia. The HR was slower than that before propranolol administration. Compared with 1st hour after propranolol initiation, the average HR was slower at 2nd hours after oral propranolol. As shown in Fig. 1, the change trend of mean HR over time was statistically significant (P < 0.005).

#### BG

Before treatment, 4 infants were found to have hypoglycemia, but all of them were clinically asymptomatic; In addition, the fast glycemia maintained within normal range after a repeat fasting plasma glucose test. During the hospitalization period, 5 patients occurred asymptomatic hypoglycemia. There was no significant change in blood glucose level before and 2 h after taking drug, as shown in Fig. 1.

### Monitoring during follow-up

#### ECG and UCG

A higher incidence of bradycardia was identified with the longer duration of propranolol treatment, while the risk of prolonged PR interval did not increase with the prolongation of treatment (Table 2). No patients were detected abnormal UCG findings due to propranolol administration during treatment.

**Table 2:** Incidence of bradycardia and PR interval prolongation.

Time (month)	Bradycardia (%)	Prolonged PR interval (%)
1	0/112 (0.0)	3/112 (2.8)
2	1/112 (0.8)	4/112 (3.5)
3	4/112 (3.6)	4/112 (3.5)
5	5/112 (4.5)	5/112 (4.5)
7	5/101 (5.0)	5/101 (5.0)
10	6/90 (6.7)	3/90 (3.3)
13	3/42 (7.1)	2/42 (4.8)

#### BG

**Figure 2:**
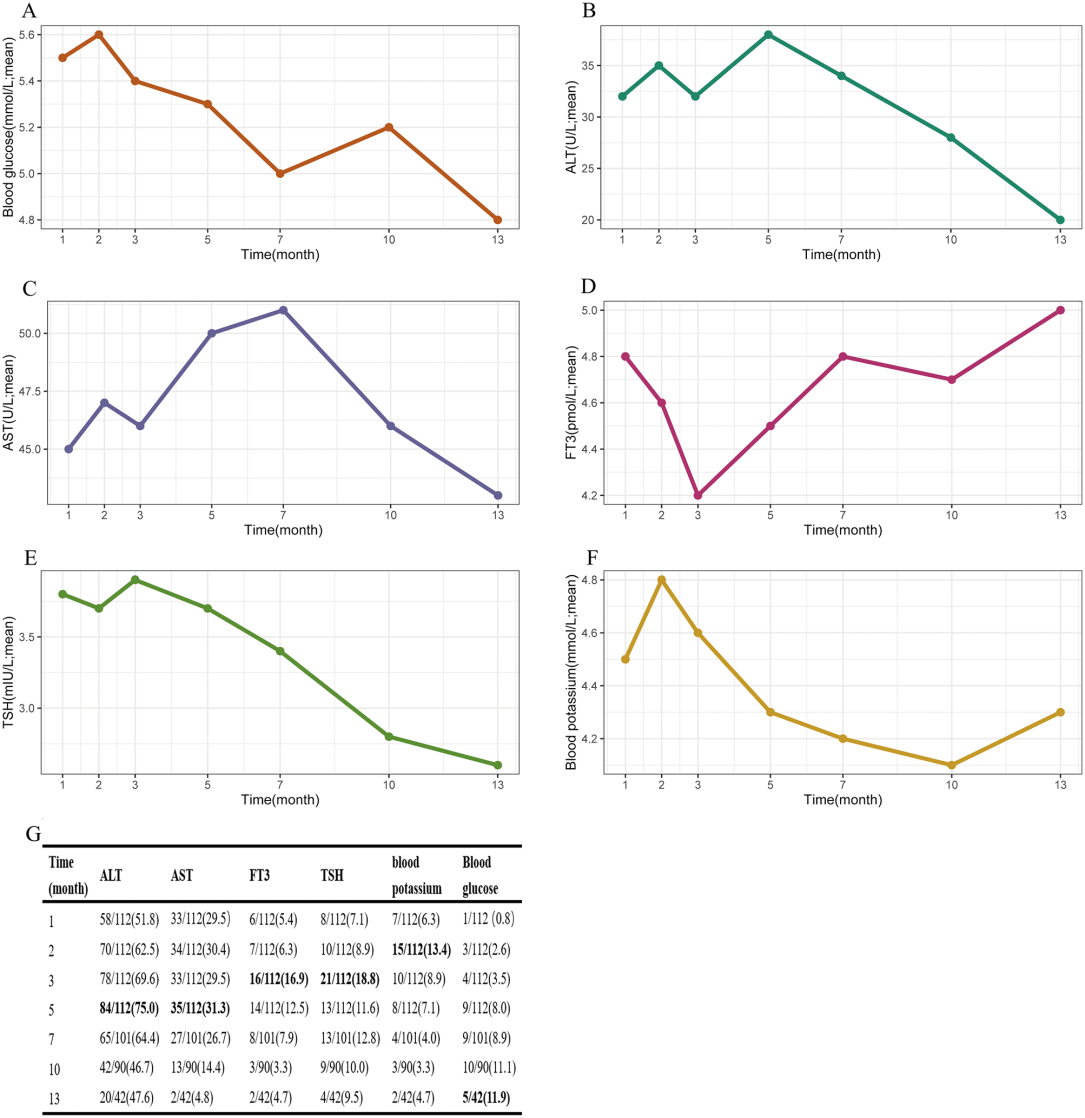
Changes of laboratory examination after propranolol during the outpatient follow-up; Change in mean blood glucose (**A**); Change in mean ALT and AST (**B,C**); Change in mean FT3 and TSH (**D,E**); Change in mean blood potassium (**F**); Abnormal laboratory test values in children during follow-up: bold values indicate the month with the highest incidence of abnormal values (**G**); *ALT* alanine transaminase; *AST* aspartate aminotransferase; *FT3* free triiodothyronine; *TSH* thyroid stimulating hormone.

The incidence of asymptomatic hypoglycemia increased with the prolongation of propranolol treatment (Fig. 2). The prevalence of hypoglycemia was 4.5% (5/112), 2.6% (3/112), 3.5% (4/112), 8.0% (9/112), 8.9% (9/101), 11.1% (10/90), 11.9% (5/42), 13.0% (3/23) and 16.6% (2/12) after 1, 2, 3, 5, 7, 10, 13, 16 and 19 months of treatment. The changing trend in blood glucose with time was significant, as shown in Fig. 2.

#### Liver enzymes, blood potassium, thyroid function

During the 13 months follow-up, alanine transaminase (ALT) increased twice as much as the normal value for 8 times, aspartate transaminase (AST) increased twice as much as the normal value for 6 times, especially in the first 5 months. The incidence of abnormal blood potassium reached the maximum at the 2 months of treatment, while the incidence of abnormal thyroid stimulating hormone (TSH) and free triiodothyronine (FT3) reached the maximum at the 3 months of treatment. The patients’ abnormal laboratory test values after initiation are summarized in Fig. 2. The changing trends in ALT, AST, TSH, FT3, and blood potassium over time are shown in Fig. 2.

#### Height, weight, and head circumference monitoring

In this study, the physical growth index for boys and girls in Zhu Futang Practical Pediatrics was considered the reference value for the physical development of children^21^.

At the age of 6 and 12 months, the average height of boys was significantly higher relative to the reference interval for healthy children (P = 0.032 and P = 0.028, respectively), while the average height of girls was significantly higher at the age of 6, 12 and 18 months (P = 0.015, P = 0.035 and P = 0.026, respectively). In boys, there was no statistical difference between the mean height and normal parameters at the age of 18, 24, and 30 months (P = 0.678, P = 0.905, and P = 0.982, respectively) and in girls at the age of 24 and 30 months (P = 0.658 and P = 0.865, respectively).

At the age of 6, 12, and 18 months, the average weight of boys was significantly heavier relative to the normal reference interval (P = 0.035, P = 0.016, and P < 0.001, respectively), while the average weight of girls was significantly higher at the age of 6 and 12 months (P = 0.043 and P = 0.012, respectively). In boys, there was no statistical difference between the mean weight and normal parameters at the age of 24 and 30 months (P = 0.783 and P = 0.942, respectively) and in girls at the age of 18, 24 and 30 months (P = 863, P = 0.782 and P = 0.845, respectively).

At the age of 6 and 12 months, the average head circumference of boys was significantly larger relative to the normal reference interval (P = 0.023 and P = 0.015, respectively), while the average head circumference of girls was significantly larger at the age of 6, 12 and 18 months (P = 0.037, P = 0.035 and P < 0.001, respectively). In boys, there was no statistical difference between the mean head circumference and normal parameters at the age of 18, 24, and 30 months (P = 0.684, P = 0.745, and P = 0.326, respectively), and in girls at the age of 24 and 30 months (P = 0.563 and P = 0.645, respectively). All of these results are shown in Table 3.

**Table 3:** Height, weight and head circumference comparison in patients (n = 112).

	Age (month)	Reference value (mean ± SD)	Patients (mean ± SD)	P
Boy				
Height	6	67.70 ± 2.40	69.80 ± 3.10	0.032*
	12	75.80 ± 2.70	77.30 ± 3.03	0.028*
	18	82.00 ± 3.10	82.32 ± 2.80	0.678
	24	87.80 ± 3.50	87.82 ± 3.22	0.905
	30	92.60 ± 3.80	92.89 ± 3.32	0.982
Weight	6	8.41 ± 0.94	9.87 ± 1.21	0.035*
	12	10.05 ± 1.11	12.12 ± 1.32	0.016*
	18	11.29 ± 1.24	13.52 ± 1.34	< 0.001*
	24	12.54 ± 1.38	12.55 ± 1.29	0.783
	30	13.64 ± 1.50	13.82 ± 1.42	0.932
Head circumference	6	43.60 ± 1.30	45.72 ± 2.13	0.023*
	12	46.40 ± 1.30	48.25 ± 1.54	0.015*
	18	47.60 ± 1.30	47.80 ± 1.53	0.684
	24	48.40 ± 1.30	48.63 ± 1.12	0.745
	30	49.10 ± 1.30	49.40 ± 1.33	0.326
Girl				
Height	6	66.10 ± 2.30	68.25 ± 2.60	0.015*
	12	74.30 ± 2.70	76.35 ± 2.03	0.035*
	18	80.80 ± 3.00	82.45 ± 3.56	0.026*
	24	86.50 ± 3.50	86.64 ± 3.23	0.658
	30	91.40 ± 3.70	91.60 ± 3.93	0.865
Weight	6	7.77 ± 0.85	8.96 ± 0.72	0.043*
	12	9.40 ± 1.02	11.34 ± 1.25	0.012*
	18	10.65 ± 1.15	10.66 ± 1.37	0.863
	24	11.92 ± 1.30	12.18 ± 1.45	0.782
	30	13.05 ± 1.45	13.19 ± 1.45	0.845
Head circumference	6	42.30 ± 2.00	44.22 ± 2.23	0.037*
	12	44.90 ± 1.90	46.95 ± 1.82	0.035*
	18	46.50 ± 2.00	49.12 ± 2.03	< 0.001*
	24	47.90 ± 2.00	47.92 ± 1.72	0.563
	30	49.00 ± 2.10	49.22 ± 1.62	0.645

*P＜0.05

#### Adverse effects and tolerance

All the adverse events were documented during the medication. 33 (30.0%) patients had diarrhea, and three patients needed to rest for 7 days because of severe diarrhea before restarting treatment. 20 (17.9%) children occurred sleep disturbances. Other common adverse effects, including vomiting, agitation, and constipation, subsided spontaneously without stopping the drug (Table 4).

**Table 4:** Adverse events with oral propranolol treatment (n = 112).

Adverse events	Value (%)
Diarrhea	33 (30.0)
Sleep disturbance	20 (17.9)
Vomiting	11 (9.8)
Cool extremities	6 (5.4)
Agitation	9 (8.0)
Bronchial hyperreactivity	5 (4.5)
Constipation	7 (6.3)
Viral upper respiratory tract infection	3 (2.7)

## Discussion

Most IHs regress spontaneously and can be managed expectantly. During the ‘expectant management’ commonly chosen by pediatric clinicians about 10–15% IHs are associated with complications, such as obstruction, functional impairment, ulceration, and disfigurement which require treatment^3^. Therefore, the initiation of management for IHs should be based on the risk–benefit analysis, with early intervention in a minority of the patients, in order to minimize long-time sequelae.

Young infants are more likely than older children and adults to be intolerant of propranolol. Clinically, there is a controversy about applying propranolol to young infants. Most studies excluded infants whose corrected age was less than 5 weeks^22^. Numerous previous studies of propranolol treatment in infants younger than 1 month proved to be safe and efficient when including a short-term follow-up^16,17,23^. Unfortunately, most of these studies have limited information about laboratory tests, cardiovascular data, physical development, and adverse events. The present study presents clinical evidence containing the above information to evaluate the safety of oral propranolol in young infants younger than 30 days of corrected age, especially with a long-term follow-up.

Propranolol is a beta-blocker, which has the potential to decrease HR and partly lower BP via the negative chronotropic and inotropic effects on the heart. One prospective research performed by Ji Y. and his colleagues concluded that the decreases in mean HR and SBP were statistically significant but not mean DBP, and all young infants presented clinically asymptomatic^17^. In our study, we found that all of the infants appeared to have a decrease in BP, but remained without clinical symptoms, which is consistent with previous research. However, our results showed that the long-term effect of propranolol on SBP was less than DBP which was significantly lower than before administration. Interestingly, the SBP of infants was lower 2 h after propranolol than 1 h after propranolol initiation, but not DBP. The mean HR at 2 h after propranolol initiation was slower (2.1 ± 1.1) than that 1 h after oral propranolol. The peak effects of oral propranolol on BP and HR in infants occurred approximately 2 h after every dose^17,24^. Consistent with previous studies, SBP and HR demonstrated the same characteristics in the present study. Propranolol can slow down the conduction of atrium and atrioventricular node, which could result in prolonging PR interval and certain abnormal ECG. Grade I atrioventricular block and T wave spikes were reported to be the common side effects on the heart of oral propranolol^25^. In our results, it demonstrated that the incidence of bradycardia was increased with prolong duration of propranolol treatment, but not the risk of prolonged PR interval, which result shares the same characteristics with infants older than 30 days^26^.

Symptomatic hypoglycemia, although rare, is a potentially severe side effect of oral propranolol. The exact mechanism of the effect of propranolol on BG remains incompletely understood so far. Still, it could block catecholamine-induced lipolysis, glycogenolysis and gluconeogenesis, which may lead to hypoglycemia in infants^27^. Young infants usually have a lower glycogen store than older infants, and they mainly rely on caregivers to provide food. In our study, all of the patients were regularly fed by their parents during the treatment. Remarkably, our results confirmed that there was no statistically significant change in BG with oral propranolol during the first 3 days but changing trend with time was significant during outpatient follow-up. This result suggested that the longer the duration of oral medication, the more prone to hypoglycemia. Thus, parental education on medication schedules and frequent feeding is vital to preventing severe hypoglycemia during medication. When the utility of beta-blocker drugs including propranolol is not appropriate, patients may occur hyperkalemia. Propranolol is an inhibitor of epinephrine that is mediated via stimulation of the beta receptor to regulate blood potassium^28^. Our results showed that the risk of hyperkalemia did not increase with prolonged duration of oral propranolol.

With the prolongation of propranolol administration, the incidence of abnormal AST and ALT decreased. The reason for abnormal liver function caused by oral propranolol may be that it is eliminated by the liver and thus formed to several other metabolites^29^. Further, young infants seem more likely to have an abnormal liver function; one of the reasons may be that the metabolic rate of propranolol is slower than that of adults.

Propranolol is an effective drug in treating hyperthyroidism through inhibiting the conversion of peripheral T4 to T3^30,31^. Not surprisingly, and in accordance with it as antithyroid drugs, some children may occur abnormal thyroid function, mainly hypothyroidism, during propranolol treatment. In our study, the incidence of abnormal TSH and FT3 reached the maximum at the 3 months of treatment and then decreased.

Currently, it is unclear whether propranolol administration affects the growth and development of children. A study by Moyakine et al. confirmed that oral propranolol could not associate with developmental risk^32^. Hu et al. implied that during the treatment, the growth curve of patients dropped by more than 20%, but nearly 50% of children backed to normal levels after drug was discontinued^33^. Huo et al. indicated that oral propranolol could be considered to be safety and efficacy, and that did not harm the physical development of children who younger than 2 years and below^34^. Recently, a large retrospective study of propranolol for IH in an Asian population concluded that propranolol did not affect the growth and development of children^26^. In our results, the height, weight and head circumference of male and female infants at 6, 12, 18, 24 and 30 months were more than the normal range. This phenomenon may be because the standard reference range exists a certain discrepancy with the actual situation, particularly owing to the improvement of China’s nutrition level in the past 10 years. Remarkably, this study assessed these physical developmental indicators of children only to the age of 30 months. Therefore, larger prospective and confirmatory studies are need to provide sufficient evidence to evaluate the long-time influence of propranolol on the physical and central nervous system development of children. Interestingly, there is a significant difference between patient and normal range at 6 and 12 months about the growth indicators, but there appears to be no difference at 24 and 30 months. The reason may be related to feeding or diet, such as breastfeeding or weaning food.

Our results indicated that oral propranolol was fairly good tolerance for the treatment of IHs in young infants. When combined with two other large studies, the most frequently adverse effects of propranolol were considered to be sleep disturbances/insomnia (~ 15%), coolness of the hands and/or feet (~ 8%), agitation/ irritability (~ 8%), diarrhea (~ 5%), sleepiness (~ 5%), and decreased appetite (~ 3%)^22,35,36^. In our study, however, the most common adverse effects were diarrhea (30.0%), sleep disturbance (17.9%), vomiting (9.8%), agitation (8.0%), which is not similar to older patients. In addition, no severe side events reported when initiating and maintaining propranolol administration, and the side effects at the present study were transient and controllable. Nevertheless, we should realize that the satisfactory result demonstrated in our research was associated with the strict inclusion and exclusion criteria. All of young infants in this study had neither diseases nor conditions that could be unable to import food normally nor contraindications for the treatment with propranolol. Based on these strict inclusion and exclusion criteria, the risk of certain side effects in young infants is more likely to be decreased.

Limitations of this study include its retrospective nature (selection bias), and small sample size. Thus, prospective studies are needed and the clinical sample size in future studies needs to be increased.

## Conclusions

In brief, our results provided a reliable basis for the evaluate of the effects of propranolol on hemodynamics, blood glucose, liver function, blood potassium, thyroid function and physical development in young infants. In addition, our study showed that oral propranolol was safe and well-tolerated for treatment of IH in properly selected young infants.

## Data Availability

The datasets used or analyzed during the current study are available from the corresponding author on reasonable request.

## Abbreviations

IH: Infantile hemangioma
UCG: Ultrasound echocardiography
ECG: Electrocardiography
BP: Blood pressure
HR: Heart rate
BG: Blood glucose
FDA: The US food and drug administration
SBP: Systolic blood pressure
DBP: Diastolic blood pressure
ALT: Alanine transaminase
AST: Aspartate transaminase
TSH: Thyroid stimulating hormone
FT3: Free triiodothyronine
T4: Tetraiodothyronine
T3: Triiodothyronine
